# Riverine barrier effects on population genetic structure of the Hanuman langur (*Semnopithecus entellus*) in the Nepal Himalaya

**DOI:** 10.1186/s12862-018-1280-4

**Published:** 2018-11-01

**Authors:** Laxman Khanal, Mukesh Kumar Chalise, Tao Wan, Xuelong Jiang

**Affiliations:** 1 State Key Laboratory of Genetic Resources and Evolution, Kunming Institute of Zoology, Chinese Academy of Sciences, Kunming, 650223 Yunnan China; 2 Kunming College of Life Science, University of Chinese Academy of Sciences, Kunming, 650223 China; 3 Central Department of Zoology, Institute of Science and Technology, Tribhuvan University, Kathmandu, 44613 Nepal; 4 State Key Laboratory for Conservation and Utilization of Bio-Resources, Laboratory of Ecology and Evolutionary Biology, Yunnan University, Kunming, China

**Keywords:** Hanuman langur, Himalaya, Genetic diversity, Riverine barrier effects, Paleodistribution

## Abstract

**Background:**

Past climatological events and contemporary geophysical barriers shape the distribution, population genetic structure, and evolutionary history of many organisms. The Himalayan region, frequently referred to as the third pole of the Earth, has experienced large-scale climatic oscillations in the past and bears unique geographic, topographic, and climatic areas. The influences of the Pleistocene climatic fluctuations and present-day geographical barriers such as rivers in shaping the demographic history and population genetic structure of organisms in the Nepal Himalaya have not yet been documented. Hence, we examined the effects of late-Quaternary glacial-interglacial cycles and riverine barriers on the genetic composition of Hanuman langurs (*Semnopithecus entellus*), a colobine primate with a wide range of altitudinal distribution across the Nepalese Himalaya, using the mitochondrial DNA control region (CR, 1090 bp) and cytochrome B (CYTB, 1140 bp) sequences combined with paleodistribution modeling.

**Results:**

DNA sequences were successfully retrieved from 67 non-invasively collected fecal samples belonging to 18 wild Hanuman langur troops covering the entire distribution range of the species in Nepal. We identified 37 haplotypes from the concatenated CR + CYTB (2230 bp) sequences, with haplotype and nucleotide diversities of 0.958 ± 0.015 and 0.0237 ± 0.0008, respectively. The troops were clustered into six major clades corresponding to their river-isolated spatial distribution, with the significantly high genetic variation among these clades confirming the barrier effects of the snow-fed Himalayan rivers on genetic structuring. Analysis of demographic history projected a decrease in population size with the onset of the last glacial maximum (LGM); and, in accordance with the molecular analyses, paleodistribution modeling revealed a range shift in its suitable habitat downward/southward during the LGM. The complex genetic structure among the populations of central Nepal, and the stable optimal habitat through the last interglacial period to the present suggest that the central mid-hills of Nepal served as glacial refugia for the Hanuman langur.

**Conclusions:**

Hanuman langurs of the Nepal Himalaya region exhibit high genetic diversity, with their population genetic structure is strongly shaped by riverine barrier effects beyond isolation by distance; hence, this species demands detailed future phylogenetic study.

**Electronic supplementary material:**

The online version of this article (10.1186/s12862-018-1280-4) contains supplementary material, which is available to authorized users.

## Background

The geographical separation of populations by barriers can alter ranging behavior, prevent dispersal, and restrict gene flow [1]. Barriers influencing population connectivity can be historical or contemporary, physical or nonphysical, and natural or manmade [2]. Animal behavior, including philopatry, foraging preferences, dispersal patterns, and mate selection, etc. can also lead to reproductive isolation and disruption of gene flow, leading to changes in population genetic structure within a species [3].

Rivers are major physical barriers, with Wallace [4] initially noting their effects on the distributions of Amazonian monkeys. Wallace’s riverine hypothesis implies that major rivers significantly reduce or prevent gene flow between populations inhabiting opposing river banks, hence promoting genetic differentiation and speciation [5]. The extent of the riverine barrier effect depends on the physical characteristics of the river as well as the ecology and dispersal ability of the taxa [6, 7]. History, width, discharge, flow, and chemistry among other factors can significantly influence the way in which rivers separate species distributions and constitute physical barriers to species dispersal [5]. Large or faster rivers have been described as insurmountable barriers for terrestrial mammals, including primates, although assessment of the riverine barrier effects in primates has been mainly confined to the Amazon [5, 8, 9], Congo River basin [3, 10–12], and Madagascar [13–16]. Among larger rivers, their lower sections are considered more effective barriers than the headwaters [17].

Rivers of the Nepal Himalaya originate at an elevation of 6000–8000 m and carry snow melt southwards to the plains at elevations lower than 100 m within a north-south distance of less than 200 km [18]. The widths of the Himalayan rivers in Nepal are less than most of those viewed as impassable to primates in the Amazon and Congo basins, but are characterized by very low temperatures of snow-fed water, elevated headwaters, and strong currents due to their extreme elevational gradients. The headwaters are narrower and their currents stronger than the lower reaches, where they become broader after uniting with many tributaries in lowland Nepal. Running along the north-south axis, these rivers divide the landmass into fragments, which impede the movement of terrestrial animals. However, the genetic influence of such isolation on the fauna within this range has not yet been documented.

Primate species have been used to assess the riverine barrier hypothesis as the taxonomic boundaries of many primates coincide with major river courses [6, 8, 19], although parapatric models of speciation have been observed for some [20, 21]. The body mass and foraging behavior of primates are considered important factors influencing their ability to cross rivers [22]. Hanuman langurs (*Semnopithecus entellus*), the only colobine primate from the Nepal Himalaya (central third of the Himalayan range), occupy a wide elevational and latitudinal distribution and are likely a good model species to explore riverine barrier effects. Hanuman langurs are restricted to the Indian subcontinent and are distributed throughout most parts of India, Nepal and Sri Lanka, as well as parts of Pakistan and Bangladesh [23]. Taxonomic ambiguity at the species and subspecies level still exists due to the occurrence of many morphotypes and incongruence among various classification schemes [24–27]. Hanuman langurs occur in diverse habitats, ranging from lowland tropical forest to subalpine forests covering multiple vegetative and climatic zones in Nepal [28]. Studies on this species in Nepal are limited to feeding ecology [29, 30] and reproductive behaviors of subtropical [31, 32] and temperate troops [33, 34]. Recently, Chalise [28] described the distribution of the species and basic morphological variations among populations from various forest fragments across Nepal.

Due to the morphological variations among the isolated populations of Hanuman langurs living in complex and fragmented forest environments as well as the effects of riverine barriers, population genetic analysis was necessary. Thus, we explored i) the level of genetic diversity among Hanuman langur populations; ii) the strength and influence of riverine barriers on the population genetic structure of Hanuman langur; and, iii) the effect of Pleistocene climatic fluctuations on the demographic history of Hanuman langurs in Nepal. To investigate these issues, we collected fecal samples from wild Hanuman langur troops covering almost the entire range of the species in Nepal from < 200 m to 3800 m asl and analyzed genetic variation within the control region (CR, 1090 bp) and cytochrome B (CYTB, 1140 bp) fragments of the mitochondrial DNA. Coalescent-based molecular analyses and paleodistribution modeling can provide a robust picture of the distributional history of a species [35–37]. Therefore, the evolutionary history of the Hanuman langur revealed from molecular analyses was further established by ecological niche modeling using a maximum entropy algorithm and paleodistribution reconstruction based on the principle of niche conservatism.

## Methods

### Study area and research species

**Fig. 1:**
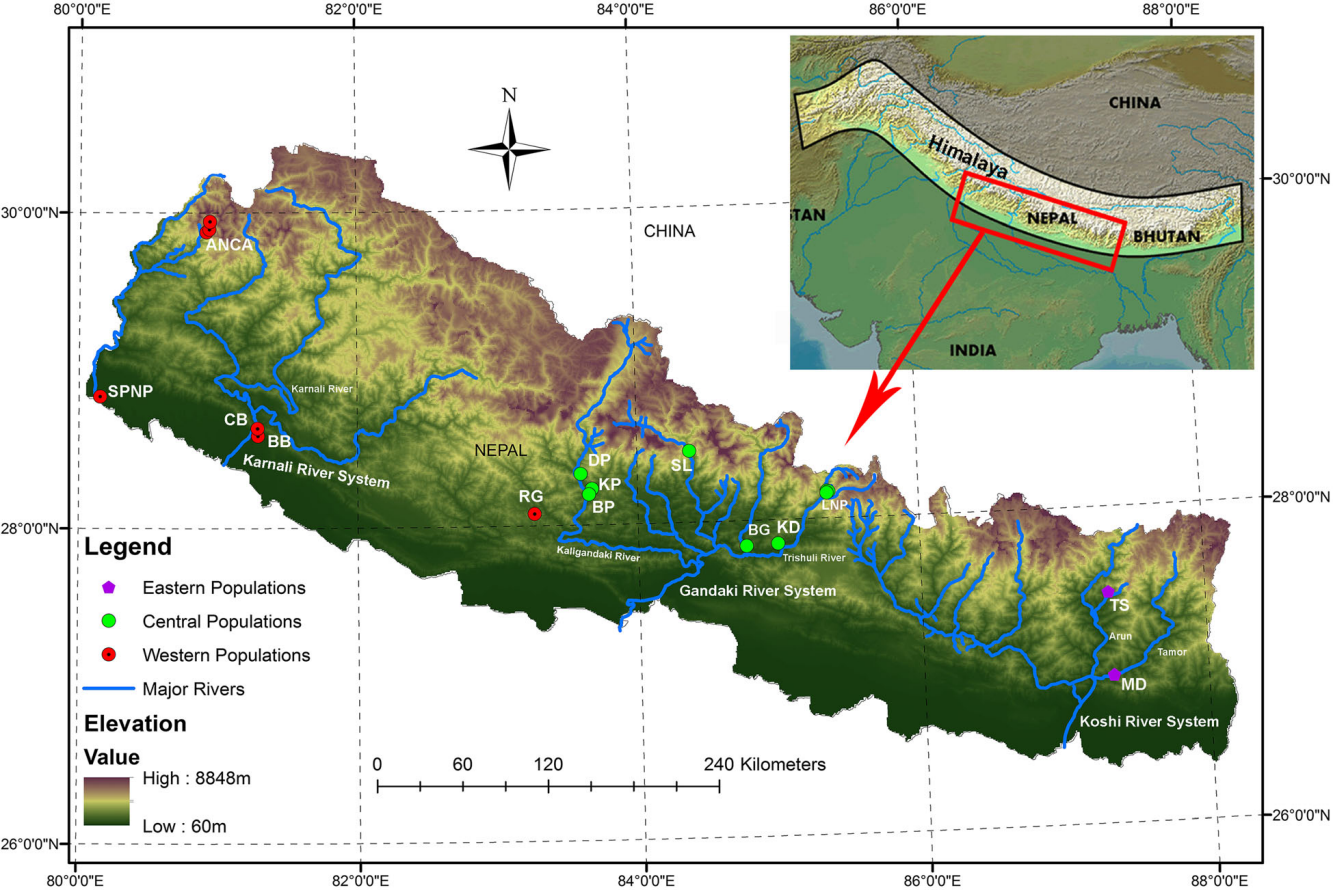
Map of study area showing the major rivers and sampling sites. Labels in the block letters represent the sampling locations, as listed in Table 1

Nepal lies on the southern flank of the central Himalaya between China and India, ranging at latitudes of 26°22′ and 30°27′ N and longitudes of 80°40′ and 88°12′ E (Fig. 1). Of the 2400 km long Himalayan range, the central one-third (~ 800 km) forms the Nepalese Himalaya (NH) [38]. Within the 200 km north-south width of Nepal, the elevation ranges from 60 m in lowland Terai to 8848 m at Mount Everest. This extreme altitudinal gradient has resulted in diverse bioclimatic zones from tropical to nival [37]. It includes the Palearctic and the Indo-Malayan biogeographical regions and hence the major floristic provinces of Asia (Sino-Japanese, Indian, western and central Asiatic, Southeast Asiatic, and African Indian desert), creating a unique and rich terrestrial biodiversity [28]. The Nepalese territory can be divided into the eastern, central, and western regions, which comprise the Koshi, Gandaki, and Karnali drainage basins, respectively [39]. Each drainage/river system has major tributaries and many minor streams that originate from the Himalayas and flow southwards.

Among the three species of nonhuman primates found in Nepal, the Hanuman langur is the only colobine, with the other two being the cercopithecine macaques (rhesus and Assam macaques) [28]. Hanuman langurs mainly distributed in the foot hills of the Nepal Himalaya and adjacent states of northern India and Pakistan [40, 41]. The Nepalese Hanuman langur populations have been classified previously as the Tarai gray langur (at lower elevations) and the Nepal gray langurs (at higher elevations) based on morphological variation. They have black faces and their pelage coloration is silver gray dorsally and white on the ventral side of the body, and highland populations bear a whorl of bright white hairs on head [28]. Their head and body length can reach up to 1 m, with a longer tail, and their body weight ranges from 7 to 20 kg, though the highland individuals are heavier than the lowland ones [42].

Hanuman langurs are diurnal, arboreal, and primarily folivorous, relying mostly on young tender leaves, buds, fruits, coniferous needles, and cones, though are known to occasionally consume termites, insect larvae [43], and even eggs of nesting birds [44]. The troops are mostly multi-male, multi-female in which adult males and females live together with young adults, juveniles, and infants. Females are philopatric. In Nepal, Hanuman langurs are found in the dipterocarp forests of outer and inner Tarai, mixed deciduous and evergreen forest of *Schima*-*Castanopsis*, *Elaeocarpus*-*Macaranga* forests in the mid-hills and mountains, and *Quercus*-*Pinus*-*Rhododendron* forests in the high mountains [42].

### Sample collection

From August 2016 to May 2017, surveys were conducted along the tributaries of the Koshi, Gandaki, and Karnali-Mahakali river systems (KRS, GRS and KMRS, respectively). In eastern Nepal, the Tamor and Arun tributaries of KRS; in central Nepal, the Trishuli, Marshyangdi and Kaligandaki tributaries of GRS; and, in far-western Nepal, the Karnali and Mahakali rivers of KMRS were sampled (Fig. 1). The surveys were carried out along the eastern and western sides of the north-south river axis starting from < 100 m asl and continuing up to 4000 m asl. Whenever a Hanuman langur troop was encountered, the occurrence point was noted using GPS, population size was estimated, and the major habitat characteristics were surveyed (Table 1).

**Table 1:** The sampling localities of *Semnopithecus entellus* and the major habitat characteristics of the habitat

Area	Symbol	Lat. (N)	Long. (E)	ASL (m)	Major habitat characteristics
Tamku, Sankhuwasabha	TS	27°27′34.64”	87°18′58.79”	556	*Shorea* forest *Albizia*, *Callicarpa*, *Helicia*, *Castanopsis*, *Ficus*, *Baliospermum*, *Alnus*, *Macaranga* and climbers
Mulghat, Dhankuta	MD	26°56′07.21”	87°19′56.14”	280	*Shorea* forest with *Bombax*, *Terminalia*, *Adina* etc.
Rishing, LNP	R-LNP	28°10′21.28”	85°21′01.94”	1950	Temperate forest with *Pinus*, *Acer*, *Magnolia*, *Quercus*, *Rhododendron* etc.
Khanjim, LNP	K-LNP	28°10′05.63”	85°21′40.75”	2508	
Shyafrubesi, LNP	S-LNP	28°09′35.82”	85°20′53.77”	1477	
Khalte, Dhading	KD	27°51′03.80”	84°59′29.30”	689	*Shorea*, *Albizia*, *Ficus*, *Eranthemum*, *Morus*, *Macaranga*, *Syzygium* etc.
Baseri, Gorkha	BG	27°50′28.15”	84°45′59.61”	382	*Shorea* forest with *Terminalia*, *Adina*, *Bombax* etc.
Saattale, Lamjung	SL	28°27′05.73”	84°22′33.25”	1533	*Schima-Castanopsis*, *Quercus*, *Toona*, *Engelhardtia*, *Ficus*, *Bombax* etc.
Kushma, Parbat	KP	28°13′37.42”	83°40′26.34”	877	Riverine broad-leaved forest *Shorea*, *Bombax*, *Anacardium* etc.
Dhairing, Parbat	DP	28°19′19.53”	83°35′43.53”	1080	Riverine broad-leaved forest with *Bombax*, *Phyllanthus*, *Wordfordia*, *Zizyphus*, *Spatholobus* and climbers
Balewa, Parbat	BP	28°11′30.56”	83°39′07.74”	685	*Shorea* forest with *Terminalia*, *Adina* etc.
Reshunga, Gulmi	RG	28°04′24.54”	83°15′42.14”	1748	Temperate broad-leaved forest with *Albizzia*, *Pinus*, *Prunus* etc.
Banke NP	BNP	28°35′09.47”	81°17′06.51”	225	Tropical *Shorea* forest with *Acacia*, *Terminalia*, *Bombax*, *Bauhinia*, *Symplocos*, *Dalbergia*, etc
Chisapani, Bardiya NP	CBNP	28°37′59.05”	81°16′57.72”	212	Tropical *Shorea* forest with *Acacia*, *Terminalia*, *Bombax*, *Bauhinia*, *Ficus*, *Dalbergia* etc.
Suklaphanta NP	SPNP	28°50′09.90”	80°09′02.28”	180	Tropical *Shorea* forest with *Acacia*, *Bombax*, *Dalbergia*, *Terminalia* etc.
Okhreni, ANCA	O-ANCA	29°52′43.80”	80°54′58.60”	2513	*Pinus*, *Quercus*, *Rhododendron*, *Betula*, *Aconitum* etc.
Dhaumula, ANCA	D-ANCA	29°53′48.90”	80°56′11.12”	3328	*Pinus*, *Quercus*, *Rhododendron*, *Alnus*, *Daphne*, *Laurel* etc.
Dhaulo Odar ANCA	DO-ANCA	29°56′41.06”	80°56′27.78”	3798	*Pinus*, *Quercus*, *Rhododendron*, *Daphne*, *Laurel* etc.

Note: *NP* National Park, *LNP* Langtang National Park, *ANCA* Api Nampa Conservation Area]

Fecal samples of wild Hanuman langurs were collected from troops that were encountered during the field surveys. A total of 87 fecal samples were collected noninvasively using sterilized cotton swabs and a plastic vial with 2 ml of lysis buffer prepared according to the protocols in White and Densmore III [45]. The dry cotton swab was rolled on the surface of the fecal sample extensively and soaked in lysis buffer to remove fecal matter and recover the mucus cells from the sample. This process was repeated three times, with similar swabbing conducted after turning the fecal sample over. To avoid re-sampling of fecal material from the same individual of a troop, the following precautions were taken: i) fecal sampling was preceded by clear identification and detailed census of the troop; ii) only fresh, moist, and intact fresh fecal material was sampled; and, iii) a troop was sampled only once from the fresh defecates after their mid-day rest. The collected samples were stored at ambient temperature and transferred to the lab for DNA extraction.

### DNA extraction

Total genomic DNA was extracted from fecal samples using the QIAamp DNA Stool Mini Kit (QIAGEN, Germany) following the manufacturer’s protocols, with some modifications: the 2 ml sample tube with lysis buffer was centrifuged for 2 min at 14000 rpm and 600 μl of the supernatant was taken for further processing; final elution was performed using 75 μl of elution buffer considering the low concentration of DNA in fecal samples.

### PCR amplification and sequencing

PCR amplification of mtDNA fragments encompassing the entire control region (CR) was performed using the primer pairs LCRF (5’-AATTGACGTTCTATCTAAACTAC-3′) and LCRR (5′- GGGGATGCTTGCATGTGTAA-3′); furthermore, the CYTB was amplified using the primer pairs LCYF (5’-CGAGATCTGAAAAACCATCGTTG-3′) and LCYR (5’-AACTGCAGTCATCTCCGGTTTACAAGA-3′). The 25 μl reaction solution contained 12.5 μl of 2× power Taq PCR Master Mix (BioTeke, Beijing), 10.5 μl of ddH_2_O, 0.5 μl of each forward and reverse primer, and 1.0 μl of template DNA. The PCR assays for loci were carried out with an initial denaturation at 94 °C for 5 min, followed by 35 cycles with denaturation at 94 °C for 30 s, annealing at 55 °C for 30 s, and extension at 72 °C for 60 s, and final extension at 72 °C for 10 min. Precautions were taken to avoid any contamination between the samples and from external sources. Only six samples were processed in one batch. Every PCR amplification was carried out with a negative control and the pre- and post-PCR work were performed independently using separate equipment.

The amplicons obtained were the intended loci and were confirmed not to be exogenous contaminants or nuclear insertions of mitochondrial DNA (numts) because: (i) colobine-specific primer pairs that successively failed to amplify high-quality vertebrate DNA were used for each locus; (ii) single PCR amplicon of the expected size was detected in all individual samples; (iii) no extreme variant sequences were detected; and iv) the coding region of CYTB had no abnormal stop codons. Moreover, as fecal samples are richer in mtDNA molecules due to their large copy numbers, small size, and lower vulnerability to degradation than the nuclear DNA, the natural degradation of DNA from fecal samples made nuclear copies even more scarce, reducing the likelihood of amplifying numts [46].

After separation in 1.2% agarose gel, the amplicons were purified and sequenced using the BigDye Terminator Cycle Kit v.3.1 (Invitrogen, USA) in both directions on an ABI 3730XL sequencer (Applied Biosystems, USA).

### Genetic data analysis

#### Genetic diversity

The CR (1090 bp) and CYTB (1140 bp) sequences were assembled and edited using Seqman (DNASTAR Lasergene v.7.1) and aligned using the Clustal W [47] algorithm in MEGA7 [48]. The sequences from respective samples were concatenated (CR + CYTB, 2230 bp) using SequenceMatrix v.1.8 [49]. Genetic diversity including number of polymorphic sites (s), haplotype number (H), haplotype diversity (Hd), and nucleotide diversity (π) were assessed separately for CR, CYTB, and CR + CYTB sequences using DnaSP v.5.10 [50]. To ensure that our analyses were comparable to other colobine research, the genetic analyses were also performed by trimming the CR sequences to leave a 489 bp long first hypervariable region (HVR I).

#### Population genetic structure

The concatenated CR and CYTB sequences were used to assess the population genetic structure. A phylogenetic tree depicting the relationship among the haplotypes was constructed using the maximum likelihood (ML) algorithm in RaxML v.8.2.10 [51], and the neighbor joining (NJ) and maximum parsimony (MP) algorithms in MEGA7 [48] with 1000 bootstrap replications. For ML analysis in RaxML, the best partitioning scheme (P1 = CYTB_1^st^, CYTB_2^nd^; P2 = CYTB_3^rd^, CR) with the GTR + G evolutionary model and Bayesian Information Criterion (BIC) was determined using Partition Finder v.2.1.1 [52]. The geographical distributions and mutational relationships of the haplotypes among the isolated populations were described by the median-joining (MJ) haplotype network constructed using PopART v.1.7 [53].

The six major clades defined by the phylogenetic tree and MJ haplotype network were considered as populations for downstream analyses. Population pairwise F_ST_ was calculated from pairwise nucleotide differences between populations and their statistical significance was checked using 10,000 permutations as implemented in Arlequin v.3.5.1.2 [54]. The genetic differentiation due to isolation by distance (IBD) was assessed by the Mantel test, which analyzed the correlation between geographic and interindividual genetic distances with 10,000 permutations executed in IBDWS v.3.23 [55]. The regression of all pairwise genetic differentiation values, F_ST_, against the corresponding log10 transformed geographical distance (in km, Additional file 1: Table S1) was used to determine the strength of the correlation. There was a high correlation (*r* = 0.876, *P* < 0.05) between the Euclidian distance and number of river tributaries separating the population pairs; therefore, the correlation results between genetic distance and number of major rivers isolating the population pairs were omitted. Further, the effect of altitudinal gradient on genetic variations was assessed by correlation between genetic distance and altitudinal gradient between the population pairs using the Mantel test in Arlequin v.3.5.1.2 [54]. Assessment of the genetic structure among the populations was performed using analysis of molecular variance (AMOVA) in Arlequin v.3.5.1.2 [54] by grouping the six populations into three geographical groups (east, central, and west) (Table 2).

**Table 2:** The DNA polymorphism and genetic diversity of isolated populations of *Semnopithecus entellus* in Nepal

Area		East	Central			West	
Population		EA	CA	CB	CC	WA	WB
Sequences (Troops)		7 (2)	17 (4)	5 (2)	13 (3)	5 (3)	20 (4)
CR (1090 bp)	S	4	14	22	10	11	25
	H	4	12	3	9	2	5
	Hd ± SD	0.810 ± 0.130	0.956 ± 0.001	0.800 ± 0.164	0.949 ± 0.042	0.600 ± 0.175	0.600 ± 0.101
	π ± SD	0.0017 ± 0.0004	0.0038 ± 0.0005	0.0119 ± 0.0031	0.0033 ± 0.0005	0.0060 ± 0.0017	0.0074 ± 0.0015
HVR1 (489 bp)	S	4	13	18	9	9	17
	H	4	12	3	8	2	5
	Hd ± SD	0.810 ± 0.130	0.956 ± 0.033	0.800 ± 0.164	0.897 ± 0.067	0.600 ± 0.175	0.600 ± 0.101
	π ± SD	0.0039 ± 0.0009	0.0081 ± 0.0012	0.0217 ± 0.0055	0.0065 ± 0.0010	0.0110 ± 0.0032	0.0112 ± 0.0022
CYTB (1140 bp)	S	2	2	24	1	5	5
	H	3	3	3	2	2	2
	Hd ± SD	0.524 ± 0.209	0.471 ± 0.118	0.800 ± 0.164	0.385 ± 0.132	0.600 ± 0.175	0.395 ± 0.101
	π ± SD	0.0005 ± 0.0002	0.0044 ± 0.0001	0.0119 ± 0.0030	0.0003 ± 0.0001	0.0026 ± 0.0007	0.0017 ± 0.0004
CR + CYTB (2230 bp)	S	6	16	46	11	16	30
	H	6	12	3	9	2	5
	Hd ± SD	0.952 ± 0.096	0.956 ± 0.033	0.600 ± 0.164	0.949 ± 0.042	0.600 ± 0.175	0.600 ± 0.101
	π ± SD	0.0011 ± 0.0002	0.0021 ± 0.0003	0.0119 ± 0.0030	0.0018 ± 0.0002	0.0043 ± 0.0012	0.0045 ± 0.0009

Note: Population symbols are the same as in Table 1; *Sequnces* Number of sequences, *Troops* Number of troops, *S* Polymorphic sites, *H* Number of haplotypes, *Hd* Haplotype diversity, π: Nucleotide diversity, *SD* Standard deviation]

#### Demographic history

Multiple complementary methods were employed to assess the demographic history of the Hanuman langurs in Nepal. Neutrality tests, Tajima’s D [56] and Fu’s Fs tests [57] in Arlequin v.3.5.1.2 [54], and the Ramos-Onsins and Rozas test (R_2_-test) [58] in DnaSP v. 5.10 [50] were performed to assess the statistical significance with 1000 permutations. Mismatch distribution analyses were performed to infer the population expansion patterns for the set of all sequences and population groups separately with 10,000 bootstrap replicates in Arlequin v.3.5.1.2 [54].

Demographic changes of Hanuman langurs through time were estimated on HVR I sequences by Bayesian skyline plot (BSP) [59] analysis in BEAST v.1.8.4 [60]. The jMODELTEST v.2.1.7 [61] was used with the Akaike information criterion (AIC) to determine the best model of nucleotide substitution (HKY + I + G). Further analyses of BSP were performed following the method in Khanal et al. [37].

#### Ecological niche modeling and paleodistribution reconstruction

Ecological niche modeling (ENM) was performed with the MaxEnt algorithm using the geographic coordinates of 29 wild Hanuman langur troops (18 troops used in molecular analyses and 11 from Chalise [28]) and bioclimatic variables. The 19 bioclimatic variables version 1.4 (Additional file 1: Table S3) for the current (~ 1950–2000) and Last Interglacial (LIG, ~ 120,000–140,000 years before present, YBP) in 30 arcsec [62] and LGM (~ 22,000 YBP) in 2.5 arcmin [63] were downloaded from the Worldclim website (http://worldclim.org/). The spatial resolutions of LGM variables were harmonized with those of current and LIG periods by resampling in 30 arcsec. All bioclimatic variables were clipped to a region from 78.5°E to 92.5°E and from 24°N to 31°N using ArcGIS 10.3.1 and exported in ASCII format. Bioclimatic variables often show high collinearity, resulting in poor model performance and misleading interpretation [64]. Therefore, correlation among the bioclimatic variables were tested by Pearson correlation test (*P* < 0.05) with the threshold of r ≥ |0.8|, and from all pairs of correlated variables beyond the threshold, only ecologically meaningful variables for the species (Bio:1, 3, 5, 11, 12, 15, 18) were selected for further analyses (Additional file 1: Table S4). MaxEnt v.3.4.1 [65] was used to model and map the current potential distribution of the Hanuman langur. The ecological niche defined by MaxEnt for Hanuman langurs was used to reconstruct their potential distributions during the period of the LGM and LIG. To facilitate the model evaluation, presence data were randomly divided into a 75% training data set and 25% validation data set, and the uncertainty introduced by such split was accounted by generating 25 replication models on a cross-validation method [62]. Area under the curve (AUC) of the receiving operating curve (ROC) was used to evaluate the accuracy of the model (Additional file 1: Figure S5). The relative importance of each environmental variable in the model was evaluated by a Jackknife test and variable contribution table.

The logistic outputs of habitat suitability for the present, LGM, and LIG were converted to binary outputs of unsuitable (0–0.377) and suitable (0.377–1) habitats using the threshold of maximum training specificity and sensitivity (maxTSS = 0.377), as explained for the model generated by presence-only data by Liu et al. [66]. The potential altitudinal shift of suitable habitats was then evaluated by overlaying binary outputs for the present, LGM, and LIG separately to the SRTM DEM (http://www.cgiar-csi.org/data/srtm-90m-digital-elevation-database-v4-1). The elevation values of suitable habitat pixels were extracted, and their mean, maximum, and minimum were computed.

## Results

### Genetic diversity

Of the 87 fecal samples analyzed, both CR (1090 bp) and CYTB (1140 bp) sequences were successfully obtained from 67 (77.01%) samples. From the CR sequences, we identified 108 polymorphic sites (S) and 35 haplotypes (H) (GenBank Accession Numbers: MH271130-MH271164) with a haplotype diversity (Hd) of 0.957 ± 0.015 and nucleotide diversity (π) of 0.02978 ± 0.0009. Most CR polymorphic sites were in the HVR I fragment (82/108), with the 67 HVR I (489 bp) sequences defining 34 haplotypes (H) with an overall Hd of 0.955 ± 0.015 and π of 0.0528 ± 0.0015. For the CYTB sequences, we identified 15 haplotypes (GenBank Accession Numbers: MH271115-MH271129) (S = 79, Hd = 0.886 ± 0.019 and π = 0.0179 ± 0.0010) and for the concatenated CR + CYTB (2230 bp) sequences we identified 37 haplotypes (S = 187, Hd = 0.958 ± 0.015, and π = 0.0237 ± 0.0008). Among the 37 haplotypes demarcated by the set of concatenated sequences, 89.19% (33/37) was troop specific, 8.11% (3/37) was shared between two troops, and 2.70% (1/37) was shared among three troops.

### Population genetic structure

**Fig. 2:**
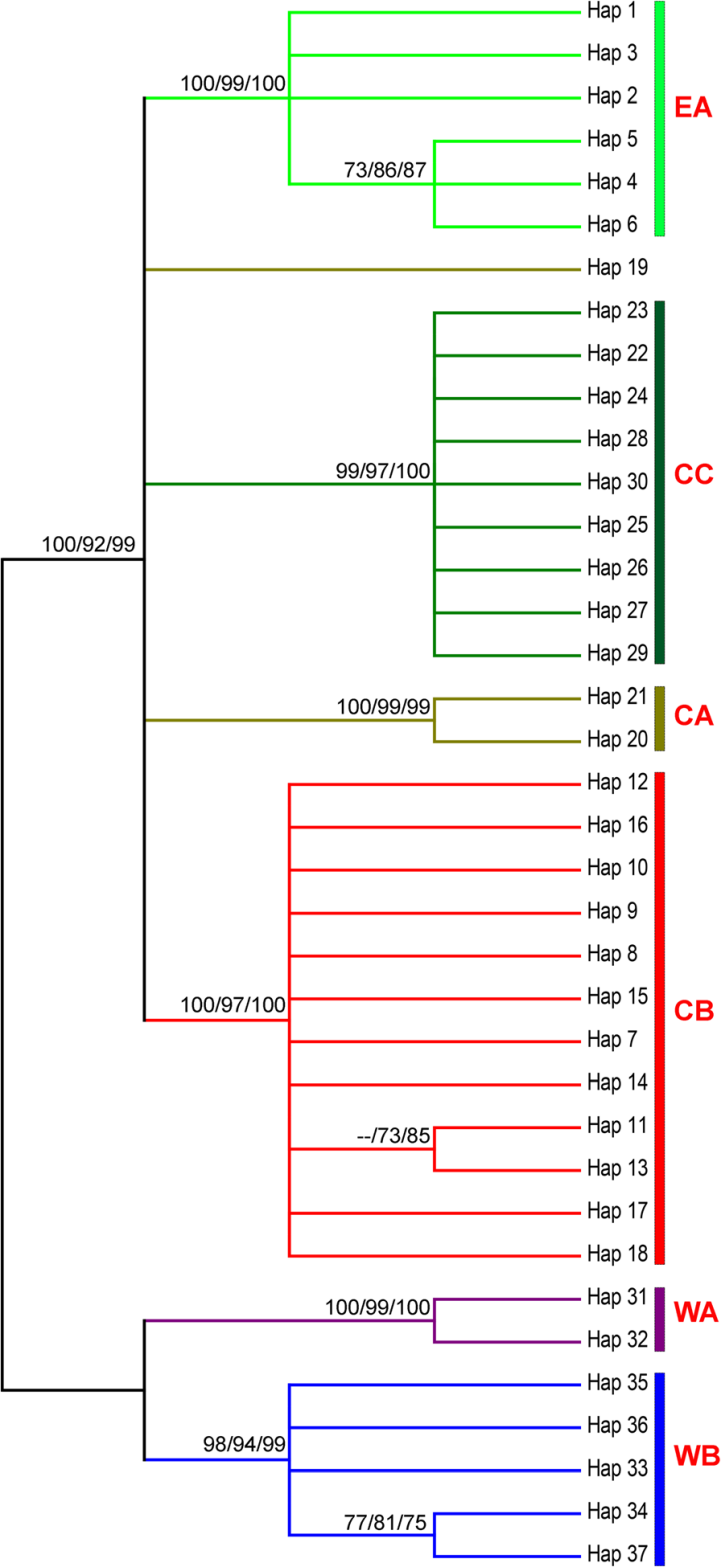
Molecular phylogeny of 37 haplotypes of *Semnopithecus entellus* from Nepal defined by CR + CYTB (2230 bp) sequences. Tip labels refer to haplotype number and three values above the branch node indicate the percentage bootstrap values (1000 replications) obtained from statistical assessments by maximum likelihood (ML), neighbor-joining (NJ) and maximum parsimony (MP) algorithms, recpectively

**Fig. 3:**
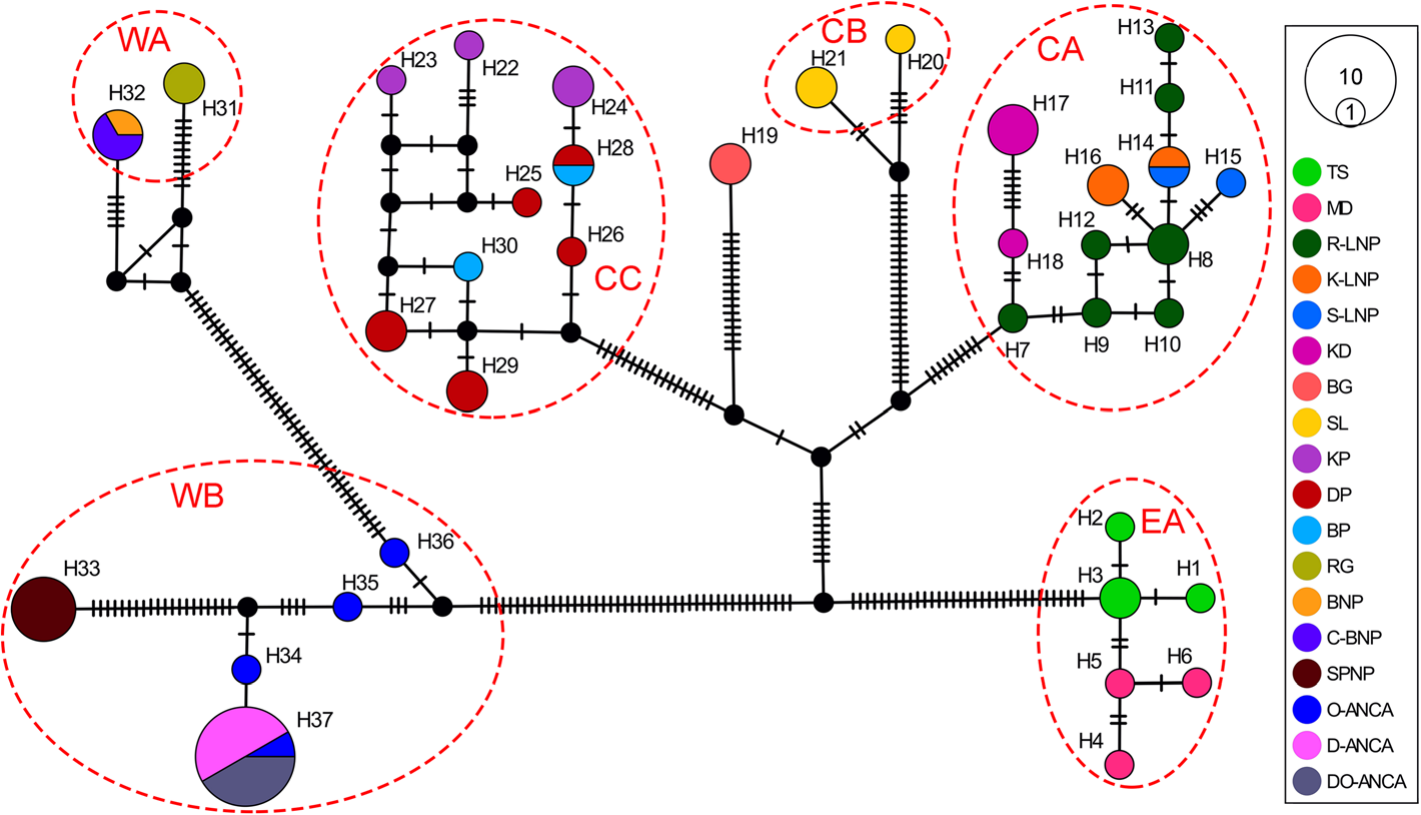
The Median joining haplotype network for 37 haplotypes of *Semnopithecus entellus* from Nepal based on CR + CYTB (2230 bp) sequences. Areas of the circles are proportional to the observed frequency of each haplotype. Shortest trees with median vectors are shown and the black-filled circles are the inferred intermediate haplotypes not sampled in this study. Each vertical dash on the lines connecting two haplotypes represents one mutational step

The gene genealogy inferred by the ML, MP, and NJ analyses consistently deduced a six-clade structure (Fig. 2). Clade I (EA) clustered all haplotypes from eastern Nepal. The sequences from central Nepal formed three major clades (CA, CB, and CC) corresponding to isolation from the Budhigandaki and Marshyangdi rivers, whereas the populations from western Nepal formed two clades (WA and WB) delineated spatially by the Karnali River. The median-joining haplotype network was consistent with the phylogenetic tree in defining six distinct clades (Fig. 3). This congruence signifies the deep divergence between the eastern, central, and western Hanuman langur populations, as well as high-level of sub-structuring in the central populations (CA, CB, and CC).

Among the six clusters (Hap_17 was included in clade CB due to its geographic proximity and closeness in the MJ network with Hap_18 and Hap_19) defined by the phylogenetic tree and MJ network, haplotype diversity (for the 2230 bp fragment) was highest in CA, whereas the nucleotide diversity was highest in CB and lowest in EA (Table 2). There were differences in sample size among the population groups but mtDNA diversity had no significant correlation with sample size (*r* = − 0.061, *P* = 0.909 for number of polymorphic sites; *r* = 0.615, *P* = 0.193 for number of haplotypes; *r* = 0.200, *P* = 0.703 for haplotype diversity; and *r* = − 0.382, *P* = 0.454 for nucleotide diversity; no r-values were statistically significant at *P* < 0.05).

**Fig. 4:**
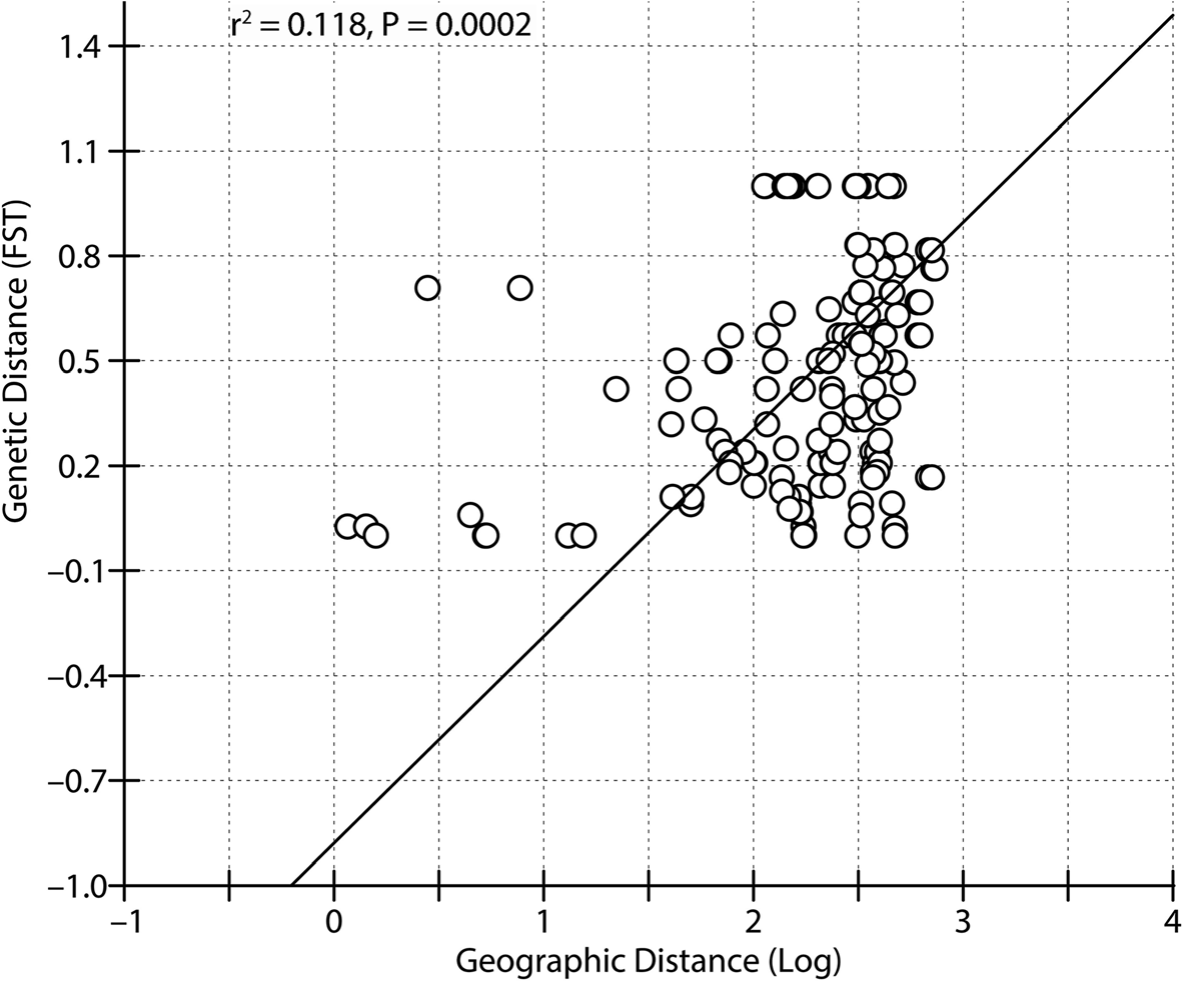
An Isolation-by-distance (IBD) analysis for *Semnopithecus entellus* of Nepal. Plot shows log transformed geographic distance along x-axis and genetic distance (F_ST_) on Y-axis

Population pairwise F_ST_ values among the six populations were high and statistically significant. The highest pairwise F_ST_ was between the EA and WA populations (0.9408) and the lowest was between the CA and CB populations (0.7246) (Table 3). The Mantel test assessing isolation by distance (IBD) revealed a statistically significant positive correlation (*r* = 0.343, *P* < 0.001) between the genetic and geographical distances among populations (Fig. 4). A similar test for weighing the effect of altitudinal gradients on genetic variation among population pairs resulted in a weaker positive correlation (*r* = 0.237, *P* < 0.05) between genetic distance and altitudinal gradient.

**Table 3:** Population pairwise F_ST_ (below diagonal), average number of pairwise differences between populations (above diagonal), and average number of pairwise differences within populations (diagonal elements in bold letters) among the sampled populations of *Semnopithecus entellus*, calculated by the distance method

	EA	CA	CB	CC	WA	WB
EA	**2.4796**	57.297	62.359	56.446	91.147	86.030
CA	0.9287	**4.7240**	38.438	35.816	81.964	74.053
CB	0.7970	0.7246	**27.072**	42.783	83.326	80.091
CC	0.9376	0.8761	0.7453	**4.0623**	80.037	77.435
WA	0.9408	0.9293	0.7794	0.9302	**9.6786**	62.393
WB	0.9053	0.8968	0.8276	0.8994	0.8391	**10.135**

All values were significant at *P* < 0.01]

Assessment of population genetic structure by AMOVA partitioned 48.00% of genetic variation among the populations within groups and 41.44% of variation among groups (Table 4). These results revealed high genetic variations among the river-isolated populations within groups and also yielded statistically significant high fixation indices suggesting a strong pattern of genetic differentiation.

**Table 4:** Analysis of molecular variance of CR + CYTB (2230 bp) sequences of *Semnopithecus entellus* in Nepal

Sources of variation	df	Sum of squares	Variance components	Percentage of variation
Among groups	2	1022.718	15.40156 Va	41.44
Among populations within groups	3	532.654	17.83838 Vb	48.00
Within populations	61	239.396	3.92452 Vc	10.56
Total	66	1794.768	37.16447	

Fixation indices: F_SC_: 0.81967; F_ST_: 0.89440; F_CT_: 0.41412; *P* < 0.01

Populations were grouped according to the Table 2 based on distribution in river systems

### Demographic history

Neutrality tests resulted in statistically nonsignificant positive values (Tajima’s D = 1.209, *P* = 0.918; Fu’s Fs = 3.493, *P* = 0.868). The Ramos-Onsin and Rozas test also yielded a high R_2_ value (R_2_ = 0.140, *P* = 0.954), indicating a relatively stable population. The neutrality test results for the individual clades were also not sufficiently statistically informative to analyze their demographic patterns (Additional file 1: Table S2).

**Fig. 5:**
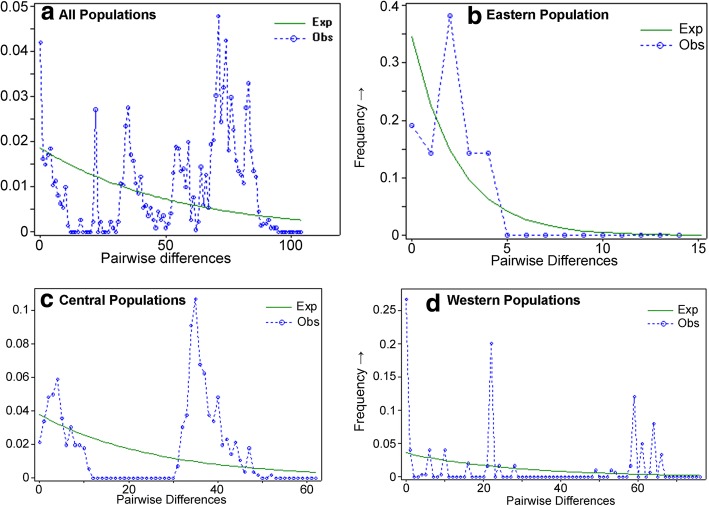
Mismatch distribution analysis inferring the demographic history of *Semnopithecus entellus* in Nepal using (**a**): entire set of sequences, (**b**): sequences from eastern populations, (**c**) sequences from central populations, and (**d**): sequences from western populations. X-axis represents the number of pairwise differences and y-axis represents the relative frequencies of pairwise comparisons

**Fig. 6:**
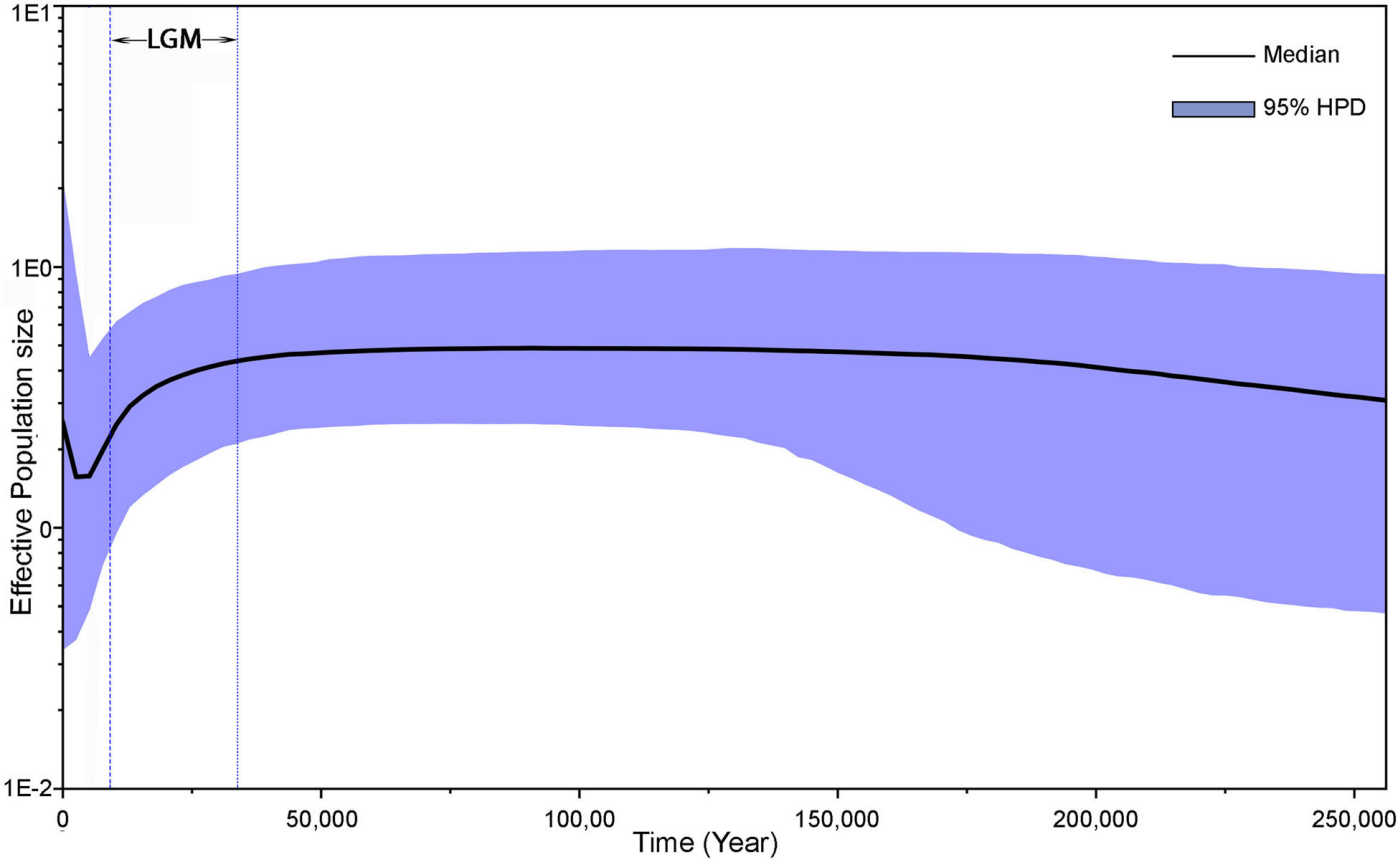
Bayesian skyline plot reconstructed using HVR I fragment (489 bp) of *Semnopithecus entellus*. X-axis is the timescale before present and Y-axis is the estimated effective population size. Solid curve indicates median effective population size; shaded range indicates 95% highest posterior density (HPD) intervals. LGM stands for the Last Glacial Maximum

The mismatch distribution analysis of the entire set of sequences projected a multimodal curve (Fig. 5) with a raggedness index (rg) of 0.0065 (*P* < 0.05). Among the population groups, the Eastern group demonstrated a unimodal curve, which peaked close to the y-axis, whereas the Central and Western groups displayed multimodal curves. The Bayesian skyline plot revealed a relatively stable effective population size (N_e_) over the last 250,000 years, though the population decreased with the onset of the LGM in the Himalayan region (Fig. 6), after which it remained stable at a lower level until the mid-Holocene, before increasing to the current population size.

### Ecological niche model and paleodistribution

Both the sampling strategies of ecological niche modeling produced similar results. In the single training/test split run, the AUC values based on training and test data were 0.934 and 0.998, respectively (Additional file 1: Figure S5). The 25 cross-validation multiplication runs resulted mean AUC of 0.892 (SD = 0.088) (Additional file 1: Figure S6). The AUC values signified that the potential distribution of Hanuman langurs fits well with our data. Annual precipitation (Bio12) had the highest contribution to the model (40.3%), whereas the contributions of precipitation seasonality (Bio15; 22.1%) and mean temperature of the coldest quarter (Bio11; 16.9%) were moderate. The response curves (Additional file 1: Figure S7) revealed that Bio12 in the range of 1600–2100 mm, Bio15 between 95 and 115, and Bio11 between 50 and 150 (5–15 °C) defined ideal Hanuman langur habitat.

**Fig. 7:**
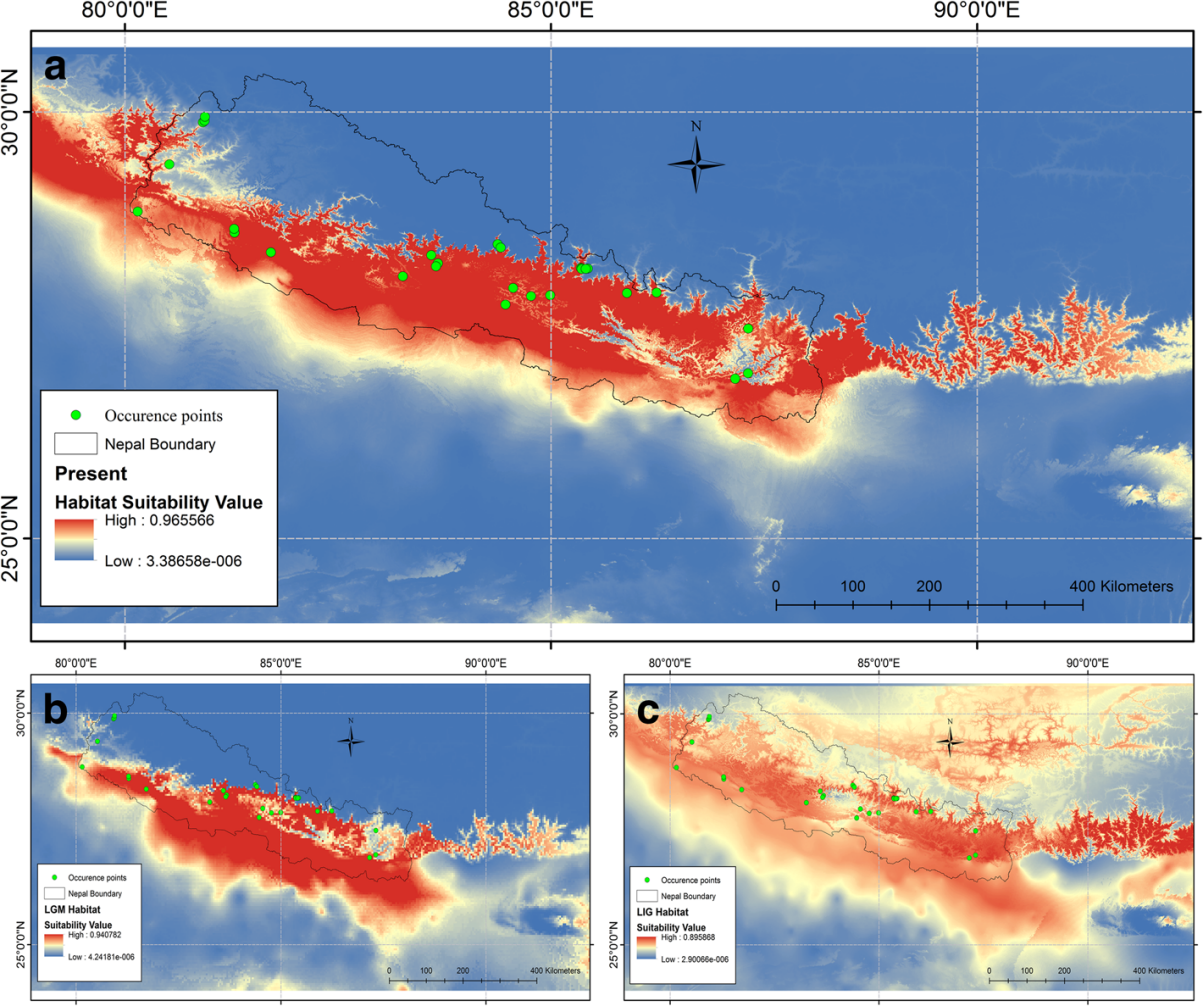
Ecological niche model projections of *Semnopithecus entellus* distribution. (**a**) Present distribution; (**b**) Potential distribution during the LGM and (**c**) Potential distribution during the LIG

According to the paleodistribution model, suitable Hanuman langur habitat at present (Max_prob = 0.965) is more extensive compared to that during the LGM (Max_prob = 0.940) and LIG (Max_prob = 0.895) (Fig. 7). Suitable habitat during the LGM almost remained the same in terms of area but shifted downward/southward from the present habitat. The current suitable habitat elevation ranges between 49 m asl to 4190 m asl, with a mean value of 1823 m asl; whereas, the historical elevational distribution of suitable habitat during the LGM and LIG ranged from 16 to 2927 m (mean = 1375 m) and 16–5356 m (mean 2435 m), respectively (Additional file 1: Figure S8). The lowland Terai and central mid-hills of Nepal were the most stable habitats from the LIG to the present day.

## Discussion

Hanuman langurs are one of the most endangered and protected non-human primate species in Nepal. The species has a wide latitudinal and elevational distribution in forest patches that are fragmented by natural barriers such as rivers and human settlements. Hanuman langurs are vulnerable to alteration and loss of their natural habitat due to anthropogenic activities, as well as conflicts with local people due to their raiding of crops [28]. One of the key elements to the management of Hanuman langurs should be a clear understanding of their population history, dynamics, and structure. Here, we used mtDNA CR (1090 bp) and CYTB (1040 bp) sequences from Hanuman langurs to explore their genetic diversity, population genetic structure, and demographic history.

### High genetic diversity

The Hanuman langur populations of Nepal demonstrated higher haplotype and nucleotide diversity (Hd = 0.955 ± 0.015 and π = 0.0528 ± 0.0015 for HVR I fragment) compared to other colobines of limited distribution range, including *Trachypithecus geei* (Hd = 0.934, π = 0.0244) from India [67], *T. leucocephalus* (Hd = 0.570 ± 0.056; π = 0.00323 ± 0.00044) [68], *Rhinopithecus roxellana* (Hd = 0.845 and π = 0.0331) [69], and *R. brelichi* (Hd = 0.457 ± 0.048, π = 0.014 ± 0.007) from China [70], but comparable to that of *R. bieti* (Hd = 0.945 ± 0.006, π = 0.036 ± 0.018) [70]. The higher genetic diversity in Hanuman langurs correlates with their morphological variations at different latitudes and elevations within Nepal [28]. According to the neutral theory of evolution, genetic diversity is proportional to effective population size and genetic variation takes a long time to accumulate in vertebrates with long generation times [71]. Thus, the observed higher genetic diversity signifies a large historical effective population size and long evolutionary history of Hanuman langurs in Nepal.

Among the populations we examined, the highest haplotype diversity was found in clade CA, which might relate to the strong elevational gradient associated with the occurrence of troops in these populations [72], even in the absence of river isolation. However, this does not explain the observation that the WB population was also sampled from a wide range of elevations but showed low haplotype diversity, even though the nucleotide diversity was higher. Intertroop haplotype sharing was very low (4/37; 10.81%) and was limited to the troops which were not isolated by rivers (H14 was shared between K-LNP and S-LNP; H28 was shared between DP and BP; H32 was shared between BNP and CBNP troops; and H37 was shared among three troops of ANCA). Such low haplotype sharing may be due to the prevention of dispersal by rivers, and the effect of female philopatry [7] as we used matrilineally inherited mtDNA for this study. Overall, the populations from central Nepal had the highest genetic diversity, and thus we hypothesized that the central populations had a comparatively longer evolutionary history and may therefore be the center of distribution [73] from which the eastern and western populations later radiated.

The overall pairwise F_ST_ between the populations was high. Population pairs in close geographical proximity had lower F_ST_ values, whereas the populations separated by distance and/or rivers had higher F_ST_ values. This F_ST_ distribution demonstrates the effectiveness of rivers as barriers as well as isolation by distance phenomenon. The genetic diversity pattern in Hanuman langurs fits the assumption of the central-abundance model that explains the reduced genetic diversity and higher genetic differentiation in peripheral populations than in central populations [74]. The average number of pairwise nucleotide differences was high between almost all population pairs but the genetic variation within populations was low, which may be due to the combined effects of smaller dispersal distance of females coupled with female philopatry and long-term isolation of populations [75].

### Riverine barrier effect and isolation by the distance

The phylogenetic inference using ML, MP, and NJ algorithms consistently defined six major clades for the Hanuman langurs in Nepal; namely EA, CA, CB, CC, WA, and WB. Clade EA from eastern Nepal and clade CA from central Nepal are isolated by the Arun, Tamakoshi, and Sunkoshi tributaries of the Koshi River system, and the Trishuli River of the Gandaki River system (Fig. 1). Clades CA and CB are isolated by the Budhigandaki and Daraundi rivers, whereas CB and CC are separated by the Marshyangdi River of the Gandaki River system. Central clades (CA, CB, and CC) are isolated from the western clades (WA and WB) by the Kaligandaki River, with its strong current, and the deep and wide Kaligandaki Gorge. The WA and WB clades from western Nepal are delineated by the Karnali and Seti rivers together with other tributaries such as the Chamelia. mtDNA phylogenies can provide unique insights into population history [76] and can indicate the boundaries of genetically divergent groups [77]. Here, the MJ haplotype network depicted the same haplotype grouping as determined by the phylogenetic analyses, and haplogroup boundaries were delineated by rivers. The network described deep divergences between eastern, central, and western populations; further, it showed sub-structuring within the central and western populations which formed three and two haplogroups, respectively. To further validate the riverine barrier effects, we grouped all sampled troops into three groups and six populations based on isolation by rivers and used AMOVA to assess the population genetic structures. Our results partitioned 48% of the genetic variations among populations within groups revealing a strong riverine barrier effect in the population genetic structure of Hanuman langur in Nepal. In addition, the genetic variation among groups (41.44%) was high, signifying isolation by distance. Similar roles of rivers in shaping the boundaries of regional haplogroups/taxa have been described for other primates such as gorillas [6], bonobos [11], lemurs [16], and tamarins [9]. The shy behavior and strong female philopatry of Hanuman langurs [42], and fast currents and cold snow-fed waters of the Himalayan rivers, with headwaters much above the elevational range of the species, have likely prevented the Hanuman langurs from crossing the rivers; however, their climatic tolerance did not prevent their distribution along extensive altitudinal gradients.

In addition to the riverine barrier effect on the population genetic structure of Hanuman langurs, isolation by distance also showed a profound effect. The nonparametric Mantel test demonstrated a statistically significant positive correlation (*r* = 0.343, *P* < 0.001) between the inter-individual genetic distance F_ST_ and the respective geographic distance, but a lower correlation (*r* = 0.237, *P* < 0.05) between the altitudinal gradients and genetic distance. Isolation by distance can explain the increase in genetic differentiation at neutral loci with geographic distance [78], with considerable time is required for this pattern to be established and stabilized [79]; the moderate correlation coefficient of Hanuman langurs may be due to its long generation time and the socio-ecological phenomenon of population fragmentation [80].

### Effects of Pleistocene climatic fluctuation on demographic history

Multiple complementary methods were used to infer the demographic history of Hanuman langurs in Nepal, which indicated long-term population stability, with some oscillations under the influence of Pleistocene climatic fluctuations. The neutrality test results did not reject the population stability but lacked statistical significance. The MDA curve was multimodal, which also signified a relatively stable population with periodic fluctuations. Both the neutrality test and MDA suggest that the population did not fluctuate to an extent sufficient to imply bottleneck or expansion [81]. The Bayesian skyline plot, which is used to estimate population dynamics of a species through time [59], indicated that the Hanuman langur population remained stable for a long period, but decreased with the onset of the LGM about 22,000 YBP. During the early-Holocene, the local LGM (LLGM) continued in the Himalayan region [82], during which the smaller Hanuman langur population remained stable. After the LLGM, with the onset of the mid-Holocene climatic optimum around 8000 YBP, the population started increasing and reached the present-day effective population size. A similar pattern was supported by the MDA curve, which revealed a peak close to the y-axis, signifying population expansion in the recent past. However, our Bayesian skyline plot analysis has some limitations that should be taken into consideration while interpreting the results. A considerable improvement on the demographic history estimates of the Hanuman langur would have been gained by using multiple non-recombining and neutrally evolving loci [83]. In addition, in highly structured populations, separate analyses at the subpopulation level would meet the assumption of panmixia [84]; however, because of the small sample size of individual clades, we performed the Bayesian skyline plot analysis on the entire set of sequences. Bayesian skyline plots together with ecological niche modeling provide more refined insights into the evolutionary history of populations [85]; hence, we further validated our molecular analysis results with paleodistribution modeling.

Pleistocene climate change and tectonic instability formed a unique scenario of disturbance ecology for the Nepal Himalaya [86]. Palynological and geomorphological data indicate the presence of dry and cold climates in higher altitudes of Nepal during the glacial periods [39]. The paleodistribution modeling during this study revealed an altitudinal/latitudinal range shift of suitable Hanuman langur habitat downward/southward during the dry and cold LGM period. Annual precipitation, precipitation seasonality, and mean temperature of the coldest quarter had higher contributions in defining the suitable habitat of the Hanuman langur. Although the Hanuman langur is a habitat generalist species with wide range of distribution [28, 87], precipitation and temperature were the powerful limiting factors for their high-altitude populations during the cold and dry glacial period. The lower elevations of central Nepal were comparatively warmer and may have received higher precipitation as they remained under the influence of both the South Asian summer monsoon and mid-latitude winter westerlies [18, 88], and, as Pleistocene refugia, should have provided amenable habitat for the Hanuman langur. The higher genetic diversity and complex population genetic structure of the Hanuman langur in central Nepal also suggest the area as a possible Pleistocene refugia for their historic populations [89]. The widespread Hanuman langur population in most of the physiographic zones of Nepal today may have been constrained during glaciation in the Himalayan region, causing them to retreat to lower elevations, especially in central Nepal. This shrinkage in habitat and food availability might have increased competition and survival threats, resulting in population size reduction. With the onset of the mid-Holocene climatic optimum, the potential habitat for the species likely increased, allowing for a concomitant spatial and demographic expansion of Hanuman langurs in the Nepal Himalaya. At present, suitable habitat is almost uniformly distributed along the entire length of the lower Nepalese Himalaya; hence, habitat interruption does not seem to constrain the distribution of the Hanuman langur. Rather, the major factor behind the genetic structuring of the Hanuman langur is the river barriers.

## Conclusions

The primary aims of this study were to explore the genetic diversity, population genetic structure and demographic history of the Hanuman langur in Nepal by assessing the riverine barrier effects and influence of the Pleistocene climatic fluctuations. The Hanuman langur populations in Nepal exhibited high genetic and nucleotide diversity and the population genetic structure was clearly demarcated by the rivers, thus supporting the riverine barrier effects. Himalayan rivers of narrow width but high flow rate with cold snow-fed water have remained effective barriers for the movement of the Hanuman langur for a long period, which has caused the accumulation of genetic variations into distinct clusters. The genetic data of the Hanuman langur and paleodistribution modeling demonstrate a robust signature of late-Pleistocene and Holocene climatic fluctuations, especially the LGM, in shaping their distribution, population genetic structure and demographic history. Being the first study of its kind in the Nepal Himalaya, our work provides baseline information on the highly diverse population genetic composition of Hanuman langur populations; further, our work justifies further fine-scale sampling and multi-locus population genetic and phylogenetic analyses to clarify the species’ ambiguous taxonomy.

## Additional file

Additional file 1: **Table S1.** Geographic distance matrix among the sampled Hanuman langur troops. **Table S2.** Neutrality tests and demographic history parameters of population groups of Hanuman langur in Nepal based on mtDNA HVR I (489 bp) sequences. **Table S3.** Predictor variables used in the construction of the niche models. **Table S4.** Correlation matrix among the 19 bioclimatic variables retrieved from the Worldclim website (http://worldclim.org/) after clipping to a region from 78.5°E to 92.5°E and from 24°N to 31°N. **Figure S5.** Area under curve (AUC) of the receiving operating curve (ROC) for the single training/test split run. **Figure S6.** Average area under the curve (AUC) for 25 replicates of MaxEnt runs. The red line is average value and blue bars represent ±1 standard deviation. **Figure S7.** The individual response curve of major three predictive variables to the Maxent model prediction. **Figure S8.** Distribution of suitable habitat pixels along the elevational gradient. X-axis represents the elevation gradients and y-axis represents the pixel frequency. (PDF 572 kb)

## Abbreviations

AMOVA: Analysis of molecular variance
asl: above sea level
CR: Control region
CYTB: Cytochrome B
IBD: Isolation by distance
LGM: Last glacial maximum
LIG: Last interglacial
LLGM: Local last glacial maximum
MDA: Mismatch distribution analysis
