# Supplementary material for: Riverine barrier effects on population genetic structure of the Hanuman langur (Semnopithecus entellus) in the Nepal Himalaya
Source: BMC Evol Biol. 2018 Nov 1;18:159. doi: 10.1186/s12862-018-1280-4 (PMC6211570; doi:10.1186/s12862-018-1280-4)

Additional Files for:

Riverine barrier effects on population genetic structure of the Hanuman Langur (*Semnopithecus entellus*) in Nepal Himalaya

Laxman Khanal[1, 2, 3], Mukesh Kumar Chalise[3*], Tao Wan[1] and Xuelong Jiang[1*]

[1] Kunming Institute of Zoology, Chinese Academy of Sciences, Kunming, Yunnan 650223, China

[2] Kunming College of Life Science, University of Chinese Academy of Sciences, Kunming 650223, China

[3] Central Department of Zoology, Tribhuvan University, Kathmandu 44613, Nepal

<u>Table S1</u>: Geographic distance matrix among the sampled troops of Hanuman Langur in kilometer (Km)

	TS	MD	RLNP	KLNP	SLNP	KD	BG	SL	KP	DP	BP	RG	BB	CB	SNP	OA	DA	DOA
**TS**	0.00																	
**MD**	58.38	0.00																
**RLNP**	209.18	239.20	0.00															
**KLNP**	208.02	238.06	1.16	0.00														
**SLNP**	208.87	238.59	1.42	1.58	0.00													
**KD**	233.34	252.81	50.26	50.67	49.11	0.00												
**BG**	254.98	272.90	68.23	68.87	67.29	22.17	0.00											
**SL**	309.52	336.76	100.47	101.63	100.71	90.13	78.04	0.00										
**KP**	368.54	388.52	164.59	165.67	164.44	136.05	115.63	73.14	0.00									
**DP**	378.46	399.55	172.91	174.02	172.84	146.79	126.78	77.75	13.09	0.00								
**BP**	369.81	389.15	166.66	167.73	166.47	136.97	116.27	76.56	4.47	15.53	0.00							
**RG**	405.17	421.39	205.37	206.40	205.09	171.87	150.21	117.04	43.93	42.87	40.53	0.00						
**BB**	605.72	623.28	400.80	401.91	400.77	372.62	351.41	302.64	237.29	228.01	235.92	201.90	0.00					
**CB**	606.93	624.95	401.58	402.70	401.57	373.96	352.84	303.12	238.41	228.93	237.13	203.63	5.25	0.00				
**SNP**	719.71	737.47	513.97	515.10	513.99	486.75	465.59	414.95	351.18	341.56	349.92	316.09	114.21	112.79	0.00			
**OA**	680.50	708.36	471.51	472.68	471.90	456.89	438.55	371.89	325.29	312.94	325.82	304.22	148.31	143.17	137.81	0.00		
**DA**	679.45	707.50	470.50	471.67	470.90	456.15	437.89	370.96	324.80	312.41	325.37	304.08	149.81	144.65	140.56	2.80	0.00	
**DOA**	681.04	709.47	472.20	473.36	472.61	458.36	440.24	372.82	327.46	315.01	328.09	307.28	154.90	149.73	145.29	7.72	5.34	0.00

Table S2: Neutrality tests and demographic history parameters of population groups of Hanuman Langur in Nepal based on mtDNA HVR I (489 bp) sequences.

**Population Groups**	**Neutrality Tests**			**Mismatch Distribution Analysis (MDA)**				
	**Tajima's D**	**Fu's Fs**	**R_2**	**SSD**	**PSSD**	**rg**	**Prg**	**Tau**
Overall	1.2091	3.4934	0.1407	0.0068	0.388	0.0065	0.009	75.406
Eastern (EA)	0.7967	-0.2260	0.2205	0.029	0.822	0.133	0.846	4.248
Central A (CA)	-0.0406	-5.0927	0.1408	0.017	0.557	0.025	0.596	4.134
Central B (CB)	1.7133	4.4278	0.2869	0.213	0.001	0.280	0.640	23.999
Central C (CC)	0.5506	-2.7881	0.1703	0.014	0.476	0.048	0.484	3.792
Western A (WA)	0.9014	6.3064	0.2381	0.291	0.325	0.227	0.256	5.862
Western B (WB)	0.3630	6.7501	0.1469	0.162	0.173	0.324	0.189	4.378

Table S3: Predictor variables used in the construction of the niche models

ABBREVIATION	DESCRIPTION
**BIO1**	Annual Mean Temperature
**BIO2**	Mean Diurnal Range (Mean of monthly (max temp – min temp))
**BIO3**	Isothermality (P2/P7) (* 100)
**BIO4**	Temperature Seasonality (standard deviation *100)
**BIO5**	Max Temperature of Warmest Month
**BIO6**	Min Temperature of Coldest Month
**BIO7**	Temperature Annual Range (P5–P6)
**BIO8**	Mean Temperature of Wettest Quarter
**BIO9**	Mean Temperature of Driest Quarter
**BIO10**	Mean Temperature of Warmest Quarter
**BIO11**	Mean Temperature of Coldest Quarter
**BIO12**	Annual Precipitation
**BIO13**	Precipitation of Wettest Month
**BIO14**	Precipitation of Driest Month
**BIO15**	Precipitation Seasonality (Coefficient of Variation)
**BIO16**	Precipitation of Wettest Quarter
**BIO17**	Precipitation of Driest Quarter
**BIO18**	Precipitation of Warmest Quarter
**BIO19**	Precipitation of Coldest Quarter

Table S4: Correlation matrix among the 19 bioclimatic variables retrieved from Worldclim website (http://worldclim.org/) after clipping to a region from 78.5°E to 92.5°E and from 24°N to 31°N.

Layer	1	2	3	4	5	6	7	8	9	10	11	12	13	14	15	16	17	18	19
**1**	1	0.26908	-0.64211	0.78213	0.27301	0.36429	0.06239	-0.03409	-0.00526	-0.00457	-0.58729	0.14839	-0.05505	-0.52015	0.08308	-0.6264	0.94857	0.3002	0.02297
**2**	0.26908	1	0.03292	0.30453	0.99326	0.98177	0.71097	0.62659	0.58967	0.67785	-0.46961	0.73497	0.56799	-0.58497	-0.07708	-0.60626	0.12834	0.79096	0.68069
**3**	-0.64211	0.03292	1	-0.51885	0.06626	-0.12845	0.27039	0.40958	0.36625	0.36019	0.77752	0.1882	0.45486	0.67639	-0.51148	0.61045	-0.60046	-0.29261	0.32915
**4**	0.78213	0.30453	-0.51885	1	0.30665	0.39653	0.01996	-0.07026	-0.04191	-0.03955	-0.57503	0.09532	-0.10353	-0.48848	0.22354	-0.54678	0.70113	0.37858	-0.01841
**5**	0.27301	0.99326	0.06626	0.30665	1	0.96795	0.70495	0.62855	0.5932	0.67612	-0.43248	0.72774	0.57361	-0.54169	-0.08954	-0.57059	0.14221	0.77462	0.67809
**6**	0.36429	**0.98177**	-0.12845	0.39653	**0.96795**	1	0.63279	0.52324	0.48429	0.58697	-0.60723	0.66838	0.45444	-0.69852	0.04807	-0.69331	0.20565	0.86215	0.59215
**7**	0.06239	0.71097	0.27039	0.01996	0.70495	0.63279	1	0.98323	0.9577	0.99243	-0.07179	0.99466	0.96298	-0.29839	-0.58454	-0.42698	0.00119	0.24577	0.99716
**8**	-0.03409	0.62659	0.40958	-0.07026	0.62855	0.52324	**0.98323**	1	0.97644	0.99382	0.10788	0.96457	0.99502	-0.11994	-0.68048	-0.27214	-0.06524	0.11622	0.99385
**9**	-0.00526	0.58967	0.36625	-0.04191	0.5932	0.48429	**0.9577**	**0.97644**	1	0.95777	0.10958	0.94553	0.9767	-0.11406	-0.71085	-0.29264	0.00327	0.06513	0.96682
**10**	-0.00457	0.67785	0.36019	-0.03955	0.67612	0.58697	**0.99243**	**0.99382**	0.95777	1	0.02657	0.97744	0.97978	-0.19769	-0.61664	-0.32935	-0.06151	0.20115	0.99786
**11**	-0.58729	-0.46961	0.77752	-0.57503	-0.43248	-0.60723	-0.07179	0.10788	0.10958	0.02657	1	-0.15321	0.1925	0.95677	-0.50774	0.87808	-0.42971	-0.72254	0.00062
**12**	0.14839	0.73497	0.1882	0.09532	0.72774	0.66838	**0.99466**	**0.96457**	**0.94553**	**0.97744**	-0.15321	1	0.94022	-0.36881	-0.55738	-0.50644	0.08332	0.28493	0.98609
**13**	-0.05505	0.56799	0.45486	-0.10353	0.57361	0.45444	**0.96298**	**0.99502**	**0.9767**	**0.97978**	0.1925	**0.94022**	1	-0.03649	-0.72848	-0.20028	-0.06536	0.03393	0.97942
**14**	-0.52015	-0.58497	0.67639	-0.48848	-0.54169	-0.69852	-0.29839	-0.11994	-0.11406	-0.19769	0.95677	-0.36881	-0.03649	1	-0.36533	0.91147	-0.35825	-0.71473	-0.22654
**15**	0.08308	-0.07708	-0.51148	0.22354	-0.08954	0.04807	-0.58454	-0.68048	-0.71085	-0.61664	-0.50774	-0.55738	-0.72848	-0.36533	1	-0.04042	-0.00997	0.44569	-0.62283
**16**	-0.6264	-0.60626	0.61045	-0.54678	-0.57059	-0.69331	-0.42698	-0.27214	-0.29264	-0.32935	0.77808	-0.50644	-0.20028	0.91147	-0.04042	1	-0.50064	-0.59965	-0.36401
**17**	**0.94857**	0.12834	-0.60046	0.70113	0.14221	0.20565	0.00119	-0.06524	0.00327	-0.06151	-0.42971	0.08332	-0.06536	-0.35825	-0.00997	-0.50064	1	0.11982	-0.02858
**18**	0.3002	0.79096	-0.29261	0.37858	0.77462	0.86215	0.24577	0.11622	0.06513	0.20115	-0.72254	0.28493	0.03393	-0.71473	0.44569	-0.59965	0.11982	1	0.19754
**19**	0.02297	0.68069	0.32915	-0.01841	0.67809	0.59215	**0.99716**	**0.99385**	**0.96682**	**0.99786**	0.00062	**0.98609**	**0.97942**	-0.22654	-0.62283	-0.36401	-0.02858	0.19754	1

Figure S5: The area under curve (AUC) of the receiving operating curve (ROC) for the single training/test split run.

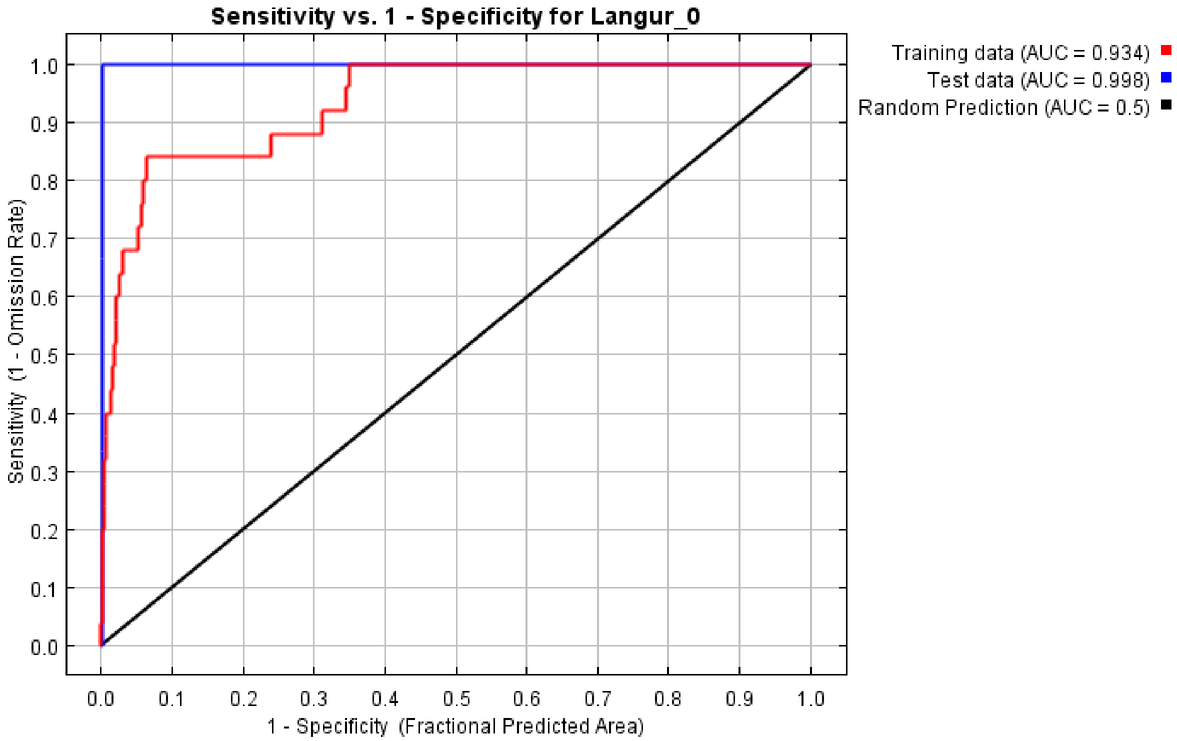

Figure S6: Average area under curve (AUC) for 25 replicates of MaxEnt runs. The red line is average value and blue bars represent plus minus one Standard Deviation.

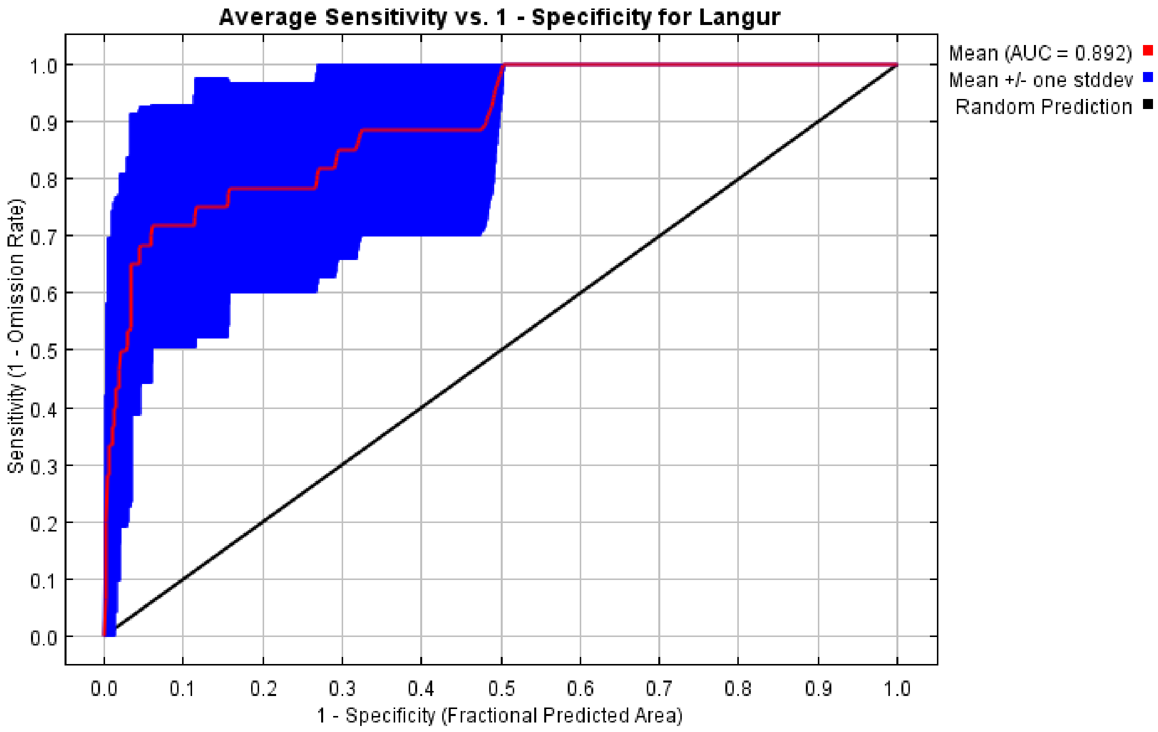

Figure S7: The individual response curve of major three predictive variables to the Maxent model prediction.

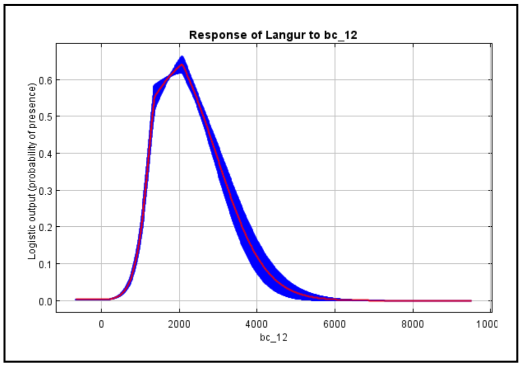

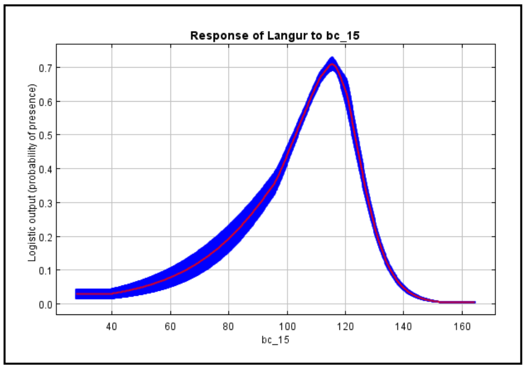

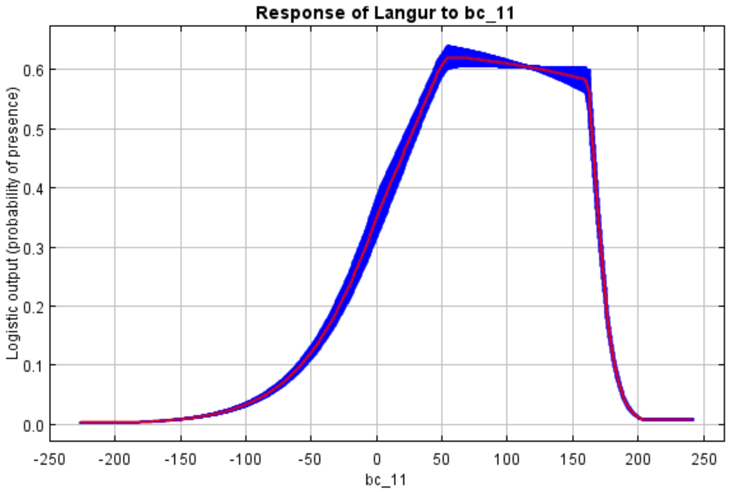

Figure S8: Distribution of suitable habitat pixels along the elevation gradient. The x-axis represents the elevation gradients and y-axis represents the pixel frequency.

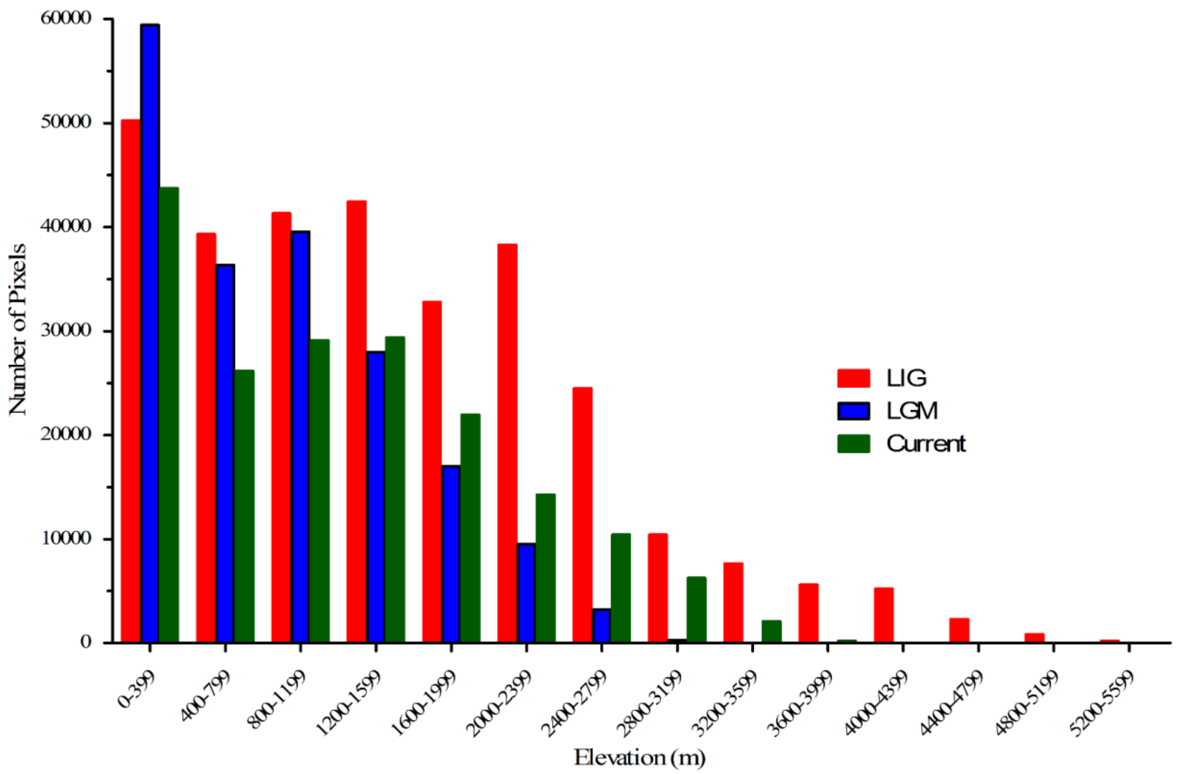

Supplement: Supplementary file 1 — Table S1. Geographic distance matrix among the sampled Hanuman langur troops. Table S2. Neutrality tests and demographic history parameters of population groups of Hanuman langur in Nepal based on mtDNA HVR I (489 bp) sequences. Table S3. Predictor variables used in the construction of the niche models. Table S4. Correlation matrix among the 19 bioclimatic variables retrieved from the Worldclim website (http://worldclim.org/) after clipping to a region from 78.5°E to 92.5°E and from 24°N to 31°N. Figure S5. Area under curve (AUC) of the receiving operating curve (ROC) for the single training/test split run. Figure S6. Average area under the curve (AUC) for 25 replicates of MaxEnt runs. The red line is average value and blue bars represent ±1 standard deviation. Figure S7. The individual response curve of major three predictive variables to the Maxent model prediction. Figure S8. Distribution of suitable habitat pixels along the elevational gradient. X-axis represents the elevation gradients and y-axis represents the pixel frequency. (PDF 572 kb) [file 12862_2018_1280_MOESM1_ESM.pdf]
